# 2346 Development of toolkits to support for researchers integrating dissemination and implementation science into their translational research

**DOI:** 10.1017/cts.2018.204

**Published:** 2018-11-21

**Authors:** Rachel Tabak, Enola Proctor, Ana A. Baumann, Alexandra Morshed, McKay V, B. Prusaczyk, D. Gerke, A. Ramsey, E. Lewis, S. Small, E. Kryzer

**Affiliations:** 1 Institute of Clinical and Translational Sciences, Washington University in St. Louis; 2 Washington University in St. Louis

## Abstract

OBJECTIVES/SPECIFIC AIMS: To use a systematic and iterative process to develop and refine toolkits to support dissemination and implementation (D&I) research. METHODS/STUDY POPULATION: Participants included research staff from the Dissemination and Implementation Research Core (DIRC), a research methods core from the Institute of Clinical and Translational Science at Washington University in St. Louis, other D&I experts from the University, and national experts from the D&I field. This project used education design research methodology and a systematic and iterative process involving several phases. The first phase (preliminary research and initial development) consisted of analysis of the educational problem and its context, and led to the development of toolkit prototypes and plans for their implementation. In the second phase (development and formative evaluation), toolkits were iteratively evaluated with emphasis on content validity and consistency and effectiveness as perceived by the users. Finally, in the summative evaluation, the toolkits were evaluated based on their use as intended. RESULTS/ANTICIPATED RESULTS: Our team identified the target audience as DIRC customers and investigators from disciplines across the University, and found that resources for beginners to D&I were lacking. The team developed 8 toolkits: (1) Introduction to D&I; (2) How to develop D&I Aims; (3) D&I Designs; (4) Implementation Outcomes; (5) Implementation Organizational Measures; (6) Assessing Barriers and Facilitators; (7) D&I Designs; and (8) Guideline research. These prototypes were iteratively revised for content validity and consistency. Finally, each toolkit was evaluated by two national experts in D&I science, and further refined. DISCUSSION/SIGNIFICANCE OF IMPACT: This systematic and cyclical process led to the development of 8 toolkits to support researchers in D&I science, which are now available on the DIRC Web site. This set the stage for development of new toolkits as additional needs are identified.

